# Epigenetic silencing and tumor suppressor gene of HAND2 by targeting ERK signaling in colorectal cancer

**DOI:** 10.1186/s12964-022-00878-4

**Published:** 2022-07-23

**Authors:** Zixu Yuan, Xihu Yu, Wenle Chen, Daici Chen, Jian Cai, Yingming Jiang, Xiaoxia Liu, Zhijie Wu, Lei Wang, William M. Grady, Hui Wang

**Affiliations:** 1 Department of Colorectal Surgery, Guangdong Provincial Key Laboratory of Colorectal and Pelvic Floor Diseases, Guangzhou, China; 2 Guangdong Institute of Gastroenterology, The Sixth Affiliated Hospital of Sun Yat-Sen University, Guangzhou, China; 3 Clinical Research Division, Fred Hutchinson Cancer Research Center, 1100 Fairview Ave N, D4-100, Seattle, WA 98109 USA

**Keywords:** HAND2, Epigenetics, Colorectal cancer, ERK signaling

## Abstract

**Background:**

The screening biomarkers for early detection of colorectal cancer (CRC) is lacking. The aim is to identify epigenetic silenced genes and clarify its roles and underlying mechanism in CRC. We conducted integrative analyses of epigenome-wide Human Methylation 450 K arrays and transcriptome to screen out candidate epigenetic driver genes with transcription silencing. Methylated silencing HAND2 were identified and verified in large CRC cohort. The mechanism of HAND2 expression by promoter inhibition were clarified both in vitro and vivo assays. Cell biofunctional roles of HAND2 methylation was investigated in CRC cells. HAND2 reconstitution were constructed by lentivirus plasmid and tumor xenograft model of HAND2 were built subcutaneously. Genomic mRNA analysis by RNA-sequencing and subsequent GSEA analysis were performed to identify potential target of HAND2 and qPCR/WB was conducted to identify the results.

**Results:**

We firstly reported high frequency of HAND2 methylation in promoter in CRC and hypermethylation was negatively correlated with expression silencing and leaded to poor survival in several CRC cohort patients. 5-Aza treatment to demethylated HAND2 could revert its expression in CRC cells. Functionally, HAND2 reconstitution can inhibit cell proliferation, invasion and migration in vitro. In tumor xenograft, HAND2 reconstruction significantly repressed tumor growth when compared to control vector. Thousands of aberrant expressed genes were observed in the heatmap of RNA-sequencing data. HAND2 reconstitution could bind to ERK and reduce its phosphorylation by CoIP assay. These above results showed HAND2 reconstitution perturbed the activation of MAPK/ERK signaling by reduction of ERK phosphorylation.

**Conclusions:**

HAND2 is one tumor suppressor by targeting ERK signaling and one potential epigenetic driver gene in CRC.

**Video Abstract**

**Supplementary Information:**

The online version contains supplementary material available at 10.1186/s12964-022-00878-4.

## Introduction

Colorectal cancer (CRC) is the third most common cancer and ranks the third of cancer death worldwide [1]. Aberrant epigenetic alterations are common hallmarks during tumorigenesis, including hypermethylation in CpG-rich sequences, also known as CpG islands in gene promotor region, and hypomethylation in global DNA region [2]. Dysregulated DNA methylation is involved in carcinogenesis, progression and maintenance in many types of cancers. DNA methylation in promoter regions can lead to silencing of mRNA transcription and decreased protein expression in many genes [3, 4]. In addition, promoter hypermethylation in a group of epigenetic driver genes are shown to be key epigenetic early events associated with cancer cell survival. Cancer cells become addicted and susceptible to aberrant DNA methylation [3]. Identification of these driver genes that occurred at the beginning of carcinogenesis will help to develop more epigenetic targeted therapies. FDA has approved a few methylated biomarkers for CRC screening test, such as promoter methylation of BMP3, NDRG4 in Cologuard kit of stool test [5], and circulating Septin 9 in Epi proColon kit in the blood. However, the sensitivity and specificity of these biomarkers for early detection of CRC are still limited, it is pivotal to find more epigenetic driver genes for potential biomarkers. Artificial intelligence (AI) is also important to diagnose CRC in our recent study published in *Annals of Surgery* [6].

Advanced epigenomic technology is now widely used in epigenome-wide analyses of cancer methylome. Thousands of aberrant DNA methylated genes in cancer are found and thus make it possible to identify cancer specific methylation and driver genes that play key roles in early carcinogenesis and cancer cell survival. Methylation levels can also be used to classify cancer subtypes and predict cancer outcomes [7]. However, distinguish these driver genes through thousands of methylated CpGs need tremendous efforts worldwide. Thus, large volumes of high throughput methylome profiles in cancer epigenome are submitted to one public database called The Cancer Genome Atlas (TCGA). Meanwhile, newly developed genome-wide mRNA sequencing technology produces big data of gene transcriptions in human cancers. It is easier to screen out potential driver genes through the integrative analysis of epigenome-transcriptome big datasets in CRC.

In this study, to identify epigenetic driver genes that are hypermethylated in the promoter along with transcription silencing, we firstly conducted integrative analyses of epigenome-wide arrays including Human Methylation 27K/450K (HM27K/450K) beadchip arrays and transcriptome including RNA sequencing datasets. About 600 patients with colon cancers from TCGA database and our own unpublished datasets are included. We have identified many candidate genes through integrate bioinformatic analysis. In this study, we focus on one most promising driver gene, heart and neural crest derivatives expressed 2 (HAND2), a transcriptional factor. Epigenetic silenced TF often regulates functional genes by promoter binding and influences downstream signaling in cancer cell survival.

Recently, Jones et al. establishes critical role of epigenetic silenced HAND2 in endometrial cancer development [8]. Hypermethylated HAND2 and silencing is early molecular event in carcinogenesis, and provide as one biomarker for early detection of endometrial cancer. However, little is known about the role of HAND2 in CRC and other cancer types. In this study, we will investigate the functional roles and molecular mechanism of HAND2 DNA methylation and epigenetic silencing in CRC. The downstream signaling of HAND2 will also be clarified by high throughput RNA sequencing and validated both in vitro and vivo assays. Novel epigenetic biomarkers for early detection of CRC and targeted therapies are potential to be developed for precision medicine.

## Results

### Aberrant methylation of HAND2 in the promoter is frequent in CRC

Firstly, we conducted epigenome-transcriptome-interactome integrated bioinformatic analyses of TCGA CRC dataset and some of our own CRC patients in Seattle lab, Washington. We have found 149 aberrant methylated CpG locus. Our focus was on these CpG locus firstly reported in CRC, located in the gene promoter region, and acted potential oncological roles. Finally, we identified one CpG cite (cg02774439) in the promoter of HAND2, which is the only methylated CpG cite of HAND2 gene.

**Fig. 1:**
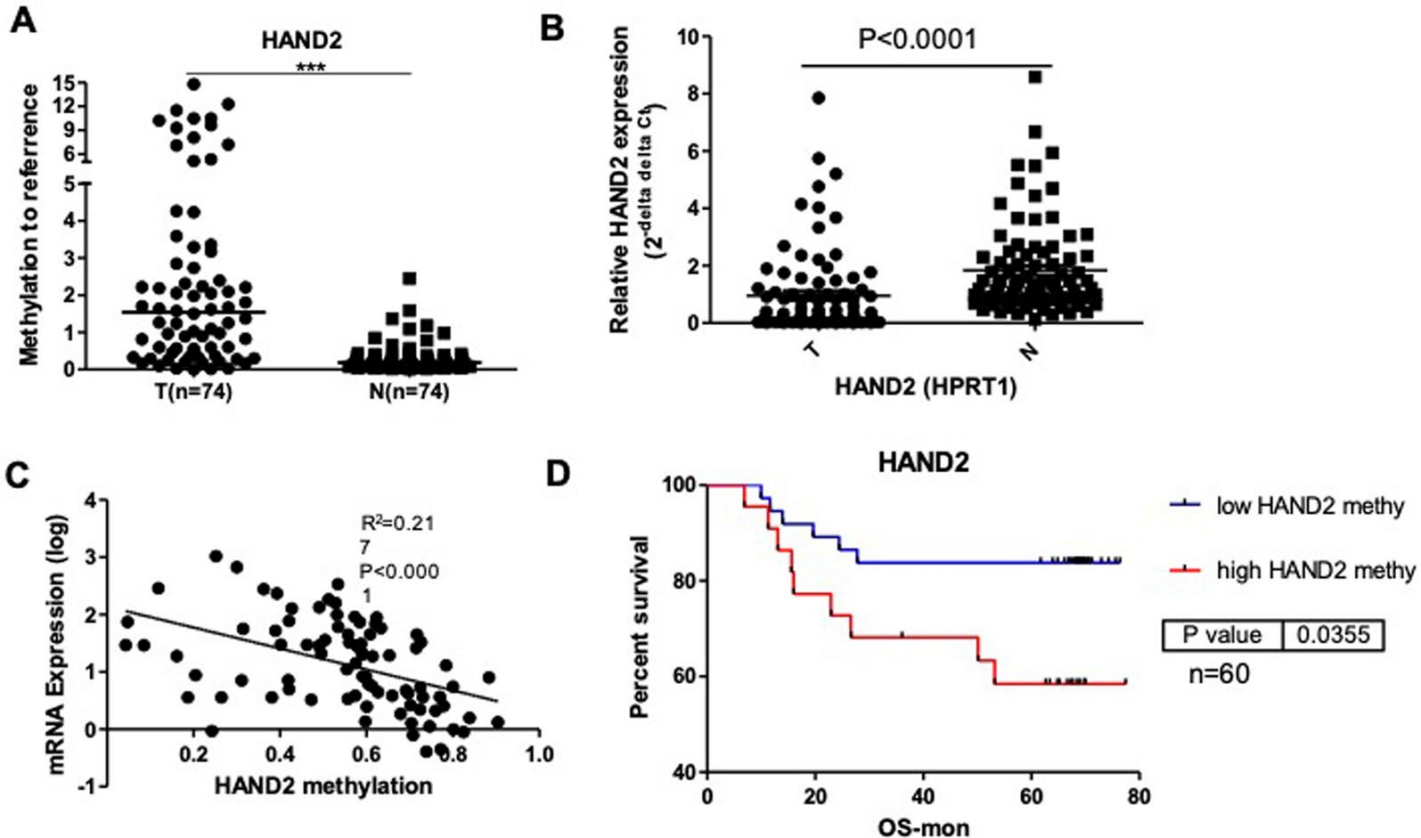
Hypermethylated HAND2 was correlated with silenced expression and predicted poor survival. **a** The methylation levels in tumor were obvious increased than paratumor normal tissue by qMSP (n = 74 pairs, paired t test, *P* < 0.001, the internal control was ALUC4). **b** The mRNA expression of HAND2 in tumor were reduced than paratumor normal tissue by qPCP (n = 86 pairs, paired t test, *P* < 0.001, the internal control was HPRT1). **c** The mRNA levels of HAND2 was negatively correlated with its methylation levels (n = 61 pairs, paired t test, r2 = 0.217, *P* < 0.0001). **d** High HAND2 methylation obtained better 5 year-overall survival (OS) than low methylation by Kaplan Meier curve (P = 0.036, n = 60)

In a Chinese cohort of CRC patients, HAND2 methylation was greatly increased in CRC tissue than paired normal tissues (tumor: 2.73% ± 3.43%; normal: 0.31% ± 0.40%; n = 74 pairs, *P* < 0.001) by qMSP (Fig. 1a). In addition, hypermethylation of HAND2 was found in 52/74 (70.3%) of CRC, which was defined as > 60% methylation by referring to endogenous control ALUC4. The methylation levels of HAND2 were at least 10 times more than paired normal tissues in 64/74 (86.5%) of tumors. In addition, the frequency of high methylation of HAND2 promoter with β value > 0.5 was found in 64% (142/223) of patients in TCGA cohort. The percent of intermediate methylation with a 0.3 < β value < 0.5 was 24% (53/223).

### Aberrant methylation of HAND2 silenced gene expression and predicted poor prognosis in CRC

To clarify the role of aberrant methylation on mRNA expression, HAND2 mRNA expression was found to be decreased dramatically in CRC tissues than paired normal tissues (n = 70 pairs, *P* < 0.001) in Chinese cohort 1 (Fig. 1b). To verify HAND2 mRNA expression in CRC, another Chinese CRC cohort 2 was measured, and HAND2 expression decreased greatly were observed in CRC tissues (0.958 ± 1.474) than paired normal tissues (1.836 ± 1.583) (n = 86 pairs, *P* < 0.001) in Additional file 1: Figure S1.

To investigate the correlation between HAND2 methylation and mRNA expression, HAND2 expression was found to be negatively correlated with its methylation (n = 61 pairs, R^2^ = 0.217, *P* < 0.001) (Fig. 1c). Furthermore, we verified this correlation in another cohort from TCGA COAD dataset. As expected, HAND2 expression was found to be negatively correlated with its methylation in TCGA cohort (n = 247 pairs, R^2^ = 0.155, *P* < 0.001) (Additional file 2: Figure S2).

To investigate the prognostic role of hypermethylated HAND2, we classified CRC patients into two groups with high or low methylation levels of HAND2 in Chinese cohort 1. The cutoff value of overall survival (OS) was decided by ROC curve. Patients with high methylation of HAND2 predicted decreased OS than patients with low HAND2 methylation (P = 0.035) (Fig. 1d).

### HAND 2 methylation repressed expression in CRC cell lines

**Fig. 2:**
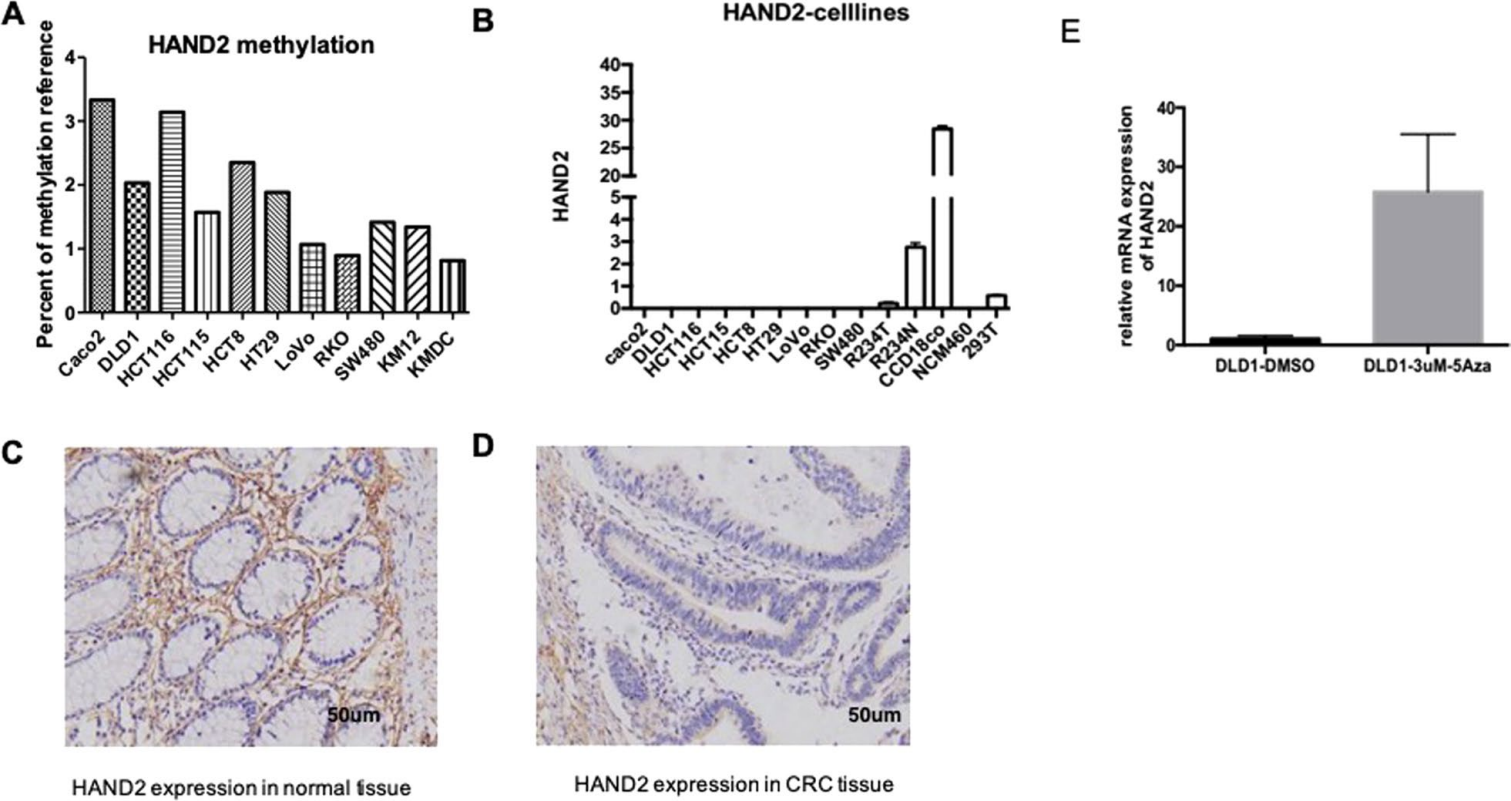
Methylated HAND2 silenced expression in CRC. **a** Most of CRC cells were presented with HAND2 hypermethylation (*P* < 0.05). **b** HAND2 mRNA expression was greatly decreased in CRC cells compared to normal colon cell CCD18co, NCM460 or normal cell 293 T of one CRC patient. Decreased HAND2 expression in the tumor tissue (R234T) when comparing to paired normal tissue (R234N) in one CRC patient for example. **c, d** The protein level of HAND2 was high in paratumor normal colon mucosa (**c**) while was decreased significant in tumor tissue (**d**) of CRC patient by IHC assay

To further investigate the effect of HAND2 methylation on expression in CRC cells, we found hypermethylated HAND2 was widely increased in almost all of CRC cell lines (Fig. 2a). The protein expression of HAND 2 in the tumor tissue was decreased than normal colon tissue of CRC patients in the Additional file 5: Figure S5. In addition, obvious decreased HAND2 expression was observed in a panel of CRC cell lines, when comparing to normal colon cells CCD18co, NCM 460 and normal cells 293 T (Fig. 2b). We then conducted IHC analysis and found decreased HAND2 protein expression in CRC tissue, when comparing to normal colon mucosa tissue (Fig. 2c, d). Interesting, we found HAND2 expression was mainly located in the colon stroma cells (Fig. 2c).

In order to determine the mechanism for loss of HAND2 expression, we found 5-Aza treatment to demethylated HAND2 and found induced HAND2 expression in DLD1 (Fig. 2e). Therefore, we concluded that the methylation of HAND2 can repress HAND2 expression. In addition, we conducted the microarray of IHC staining of HAND2 protein expression in the tumor tissue (lower expression) and normal colon tissue (higher expression) of CRC patients . HDNA2 expression is decreased in tumor tissue (Additional file 1: Figure S5).

### Methylated HAND2 promoted cell proliferation, invasion and migration

**Fig. 3:**
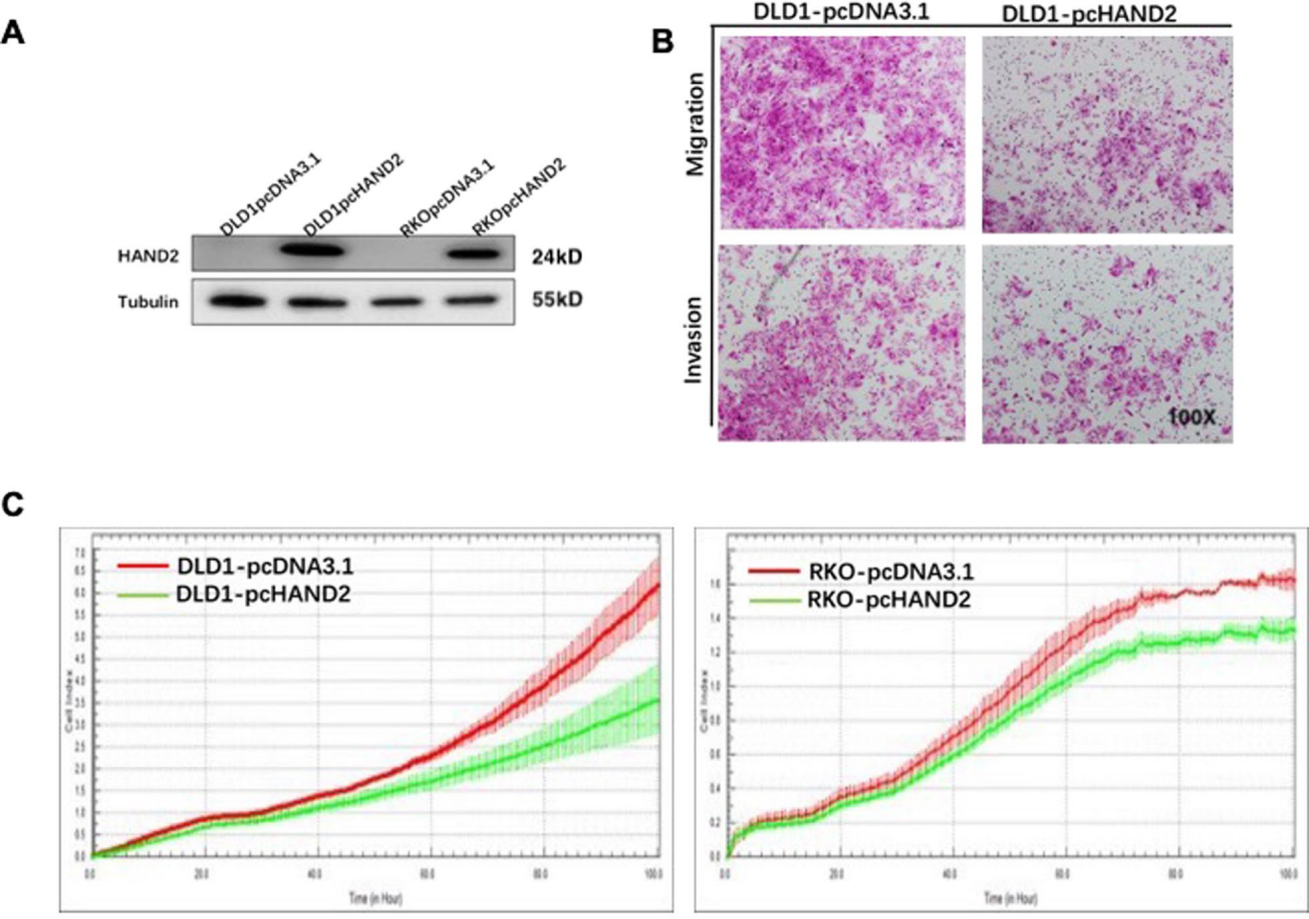
HAND2 reconstitution could repress cell proliferation, invasion and migration. **a** In DLD1 and RKO cells, HAND2 overexpressing plasmid (pcHAND2) or control vector (pcDNA3.1) were transfected with cells. Protein level of HAND2 were greatly increase after reconstitution, which revealed the successful construction of HAND2 overexpressing cells. **b** The invasion and migration capacities in DLD1 cells with HAND2 reconstitution (pcHAND2) were decreased than control cells (pcDNA3.1) by 24-well transwell assays (n = 3). **c** Cells with HAND2 reconstitution (pcHAND2) could inhibit cell proliferation than control cells (pcDNA3.1) by RTCA assay in both DLD1 and RKO cells (n = 3)

To investigate the effect of HAND2 on CRC, we constructed HAND2 overexpressed vector and transfected into RKO and DLD1 cells. In these cell lines, HAND2 protein expression by western blot was greatly recovered when compared to the transfected cells of control vector, which indicated the successful construction of HAND2 expressed cells (Fig. 3a).

Furthermore, we determined the cellular functional roles of HAND2 in CRC. Cell proliferation was greatly decreased when HAND2 was reconstituted in both DLD1 and RKO cells by RTCA assays (Fig. 3c). Cell invasion and migration capacity were also repressed by HAND2 reconstitution in DLD1 cells (Fig. 3b). However, no significant effects of cell cycle were shown when HAND2 expression was rebuilt (Additional file 3: Figure S3).

### HAND2 suppressed tumor growth in vivo

**Fig. 4:**
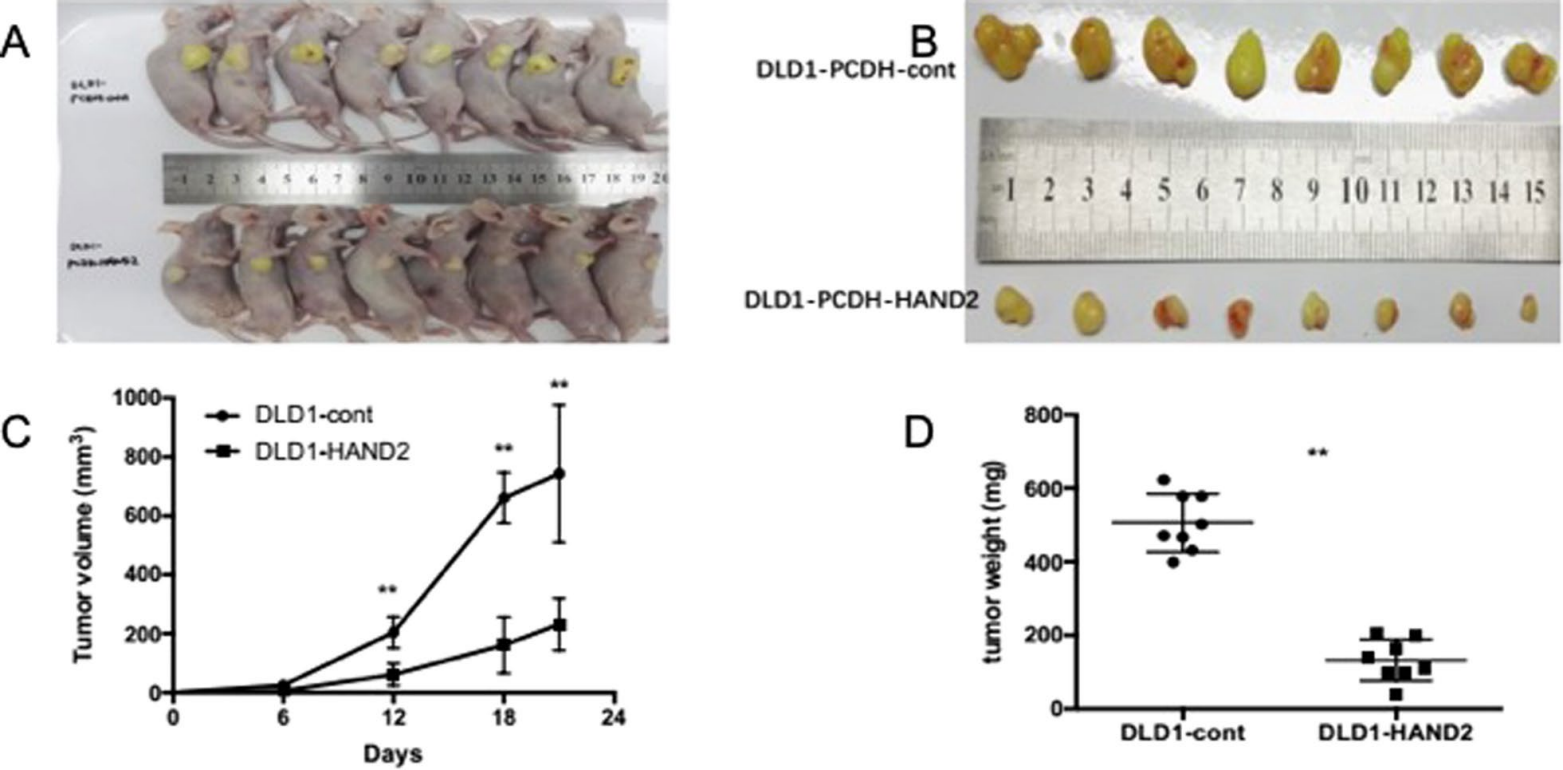
HAND2 could repress tumor growth in CRC tumor xenograft. The tumor in mice (**a**) and the size (**b, c**) and weight (**d**) of resected tumors were significantly decreased in mice with HAND2 reconstitution compared control mice (** *P* < 0.01). The mice were injected with DLD1 cells with HAND2 reconstitution (DLD1-HAND2) or control vector (DLD1-cont) and were sacrificed 12d, 18d and 24d after injection subcutaneously

We also performed studies in vivo on tumor growth and xenograft formation in constructed stable cell lines to assess the suppressor role of HAND2 in CRC. HAND2 stable cells were injected into nude mice subcutaneously. HAND2 reconstruction significantly repressed tumor growth when compared to xenografts that contained a control vector (Fig. 4a–d). 24 days after the injection of CRC cells, mice were sacrificed and the tumors were resected and measured. Both the weight and volume of HAND2-expressing tumors were greatly decreased compared to control xenografts (Fig. 4a–d). These results indicated HAND2 acted as a tumor suppressor in CRC.

### HAND2 reconstruction perturbed the activation of MAPK/ERK signaling

**Fig. 5:**
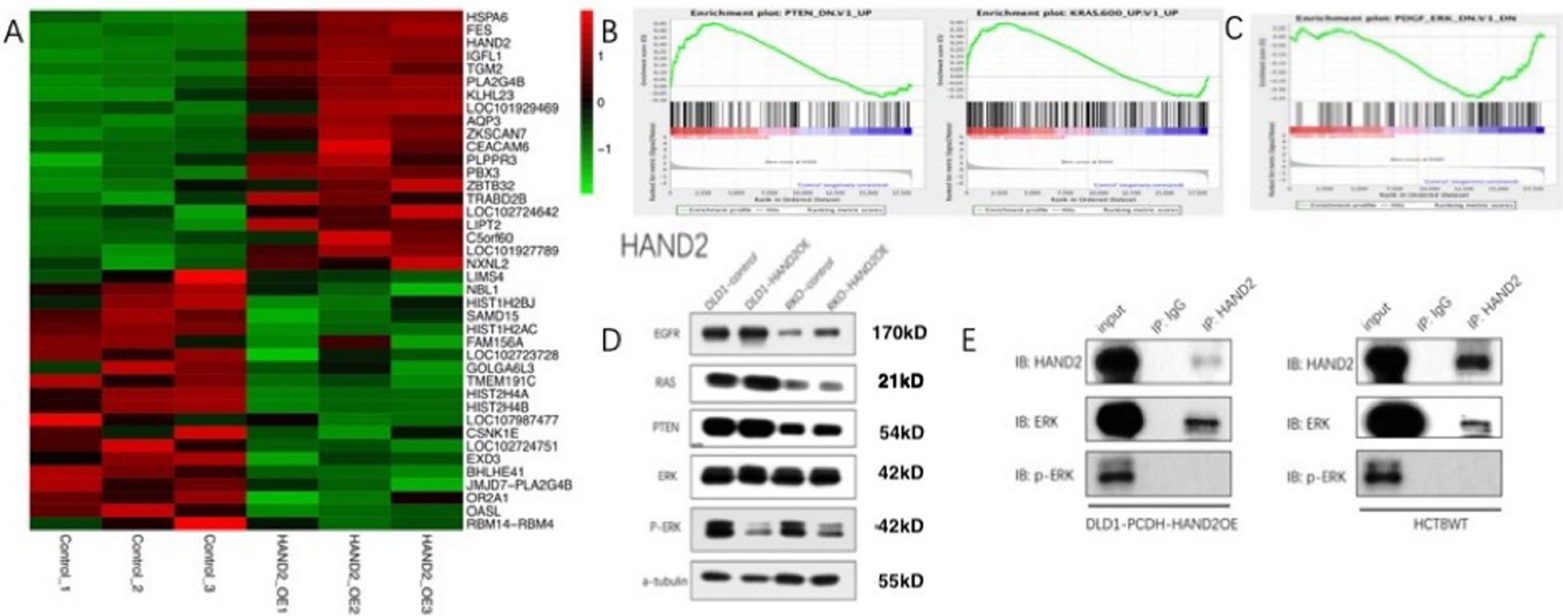
HAND2 could regulate MAPK/ERK signaling pathway by reducing phosphorylation of ERK. **a** Deep RNA sequencing were conducted to compare the mRNA expression profile between DLD1 cells with HAND2 reconstitute cells (HAND2-OE) and control vector. (Control) (n = 3). Thousands of aberrant expressed genes were identified in heatmap analysis. **b, c** Both PTEN and KRAS pathways were upregulated (**b**) while ERK signaling were downregulated (**c**) after HAND2 reconstitution by GSEA analysis of RNA-seq data. **d** Decreased P-ERK were observed in HAND2 overexpressed cells (HAND2OE), while no significant changes of EGFR, RAS, PTEN and ERK were shown in both DLD1 and RKO cells by western blot. **e** HAND2 could directly bind to ERK in both cells with HAND2 constitution (DLD1-PCDH-HAND2OE) and wild type CRC cells (HCT8WT), while the binding capacity with p-ERK was greatly decreased by CoIP assays

As shown in above experiments in vitro and vivo, HAND2 played the suppressing role in CRC. We then explore the downstream signaling pathway that were affected by HAND2. We conducted whole genomic mRNA analysis by high throughput RNA-seq in DLD1 cells with HAND2 reconstruction compared to cells with the control vector. Thousands of genes were increased or decreased in the heatmap analysis (Fig. 5a). Then we conducted GSEA analysis to determine these affected pathways. Both PTEN pathway, and KRAS pathway were upregulated, while PDGF-ERK pathway was downregulated (Fig. 5b, c). In order to confirm the RNA-seq data, we conducted western blot in cells with HAND2 reconstruction and control vector. In both DLD1 and RKO cells, decreased p-ERK levels in HAND2 overexpressed cells were observed compared to control. However, no significant changes of ERK, PTEN, RAS and EGFR levels were shown (Fig. 5d). In addition, we have also conducted CoIP assay in HAND2 reconstruction DLD1 cells and wild-type HCT8 cells. As expected, we observed HAND2 could directly bind to ERK both in DLD1 cells and HCT8 cells (Fig. 5e), which indicated HAND2 reconstitution could bind to ERK and reduce its phosphorylation for ERK inactivation to inhibit tumor growth. In addition, loss of PTEN expression in HAND2 reconstitution cells was shown in mRNA level by qPCR (Additional file 4: Figure S4), but not observed in protein level by western blot (Fig. 5d). These above results indicated the reconstruction of HAND2 perturbed the activation of MAPK/ERK signaling by reduction of ERK phosphorylation.

## Discussion

The average human genome contains 1000–3000 aberrant methylation genes in CRC [4]. High-throughput epigenome-wide arrays identify a large number of de novo methylation in gene promoter regions in CRC patients in our previous study [9]. However, it is a challenging task to discriminate epigenetic driver genes, because many aberrant methylation genes obtain no effect on mRNA expression and tumor information like “passengers” [10]. In this study, we firstly identified HAND2 through epigenome-transcriptome integrated bioinformatic analyses. High frequency of HAND2 cancer-specific methylation and silenced expression and poor survival are shown in CRC. HAND2 reconstitution can inhibit cell proliferation, invasion and migration in vitro, and also suppress CRC growth in tumor xenograft. Through RNA-sequencing assays and subsequent GSEA analysis, we found HAND2 reconstitution perturbed the activation of MAPK/ERK signaling by reduction of ERK phosphorylation. Therefore, HAND2 exhibits classical features of tumor suppressor gene in CRC.

HAND2 belongs to the basic helix-loop-helix family of transcription factors. HAND2 involves mainly biological roles such as development in ventricular chambers, cardiac morphogenesis, angiogenesis, and enteric nerve system [11], and plays anti-proliferation role in uterine epithelium [12–14]. In this study, we firstly identify methylated HAND2 with silencing expression and reveals functional roles of HAND2 in CRC through two independent Chinese cohorts and one TCGA cohort. Methylated HAND2 is even more common than other classic alterations such as *TP53, KRAS PIK3CA* and *PTEN* mutations or microsatellite instability in CRC [15, 16]. What we find here in CRC is similar to functions of HAND2 methylation in endometrial cancer. Jones et al. firstly reported HAND2 hypermethylation is common and acts as a suppressor gene in premalignant endometrial lesions and early-stage endometrial cancer [8]. These roles of HAND2 methylation indicates HAND2 is a potential cancer-specific driver gene in early carcinogenesis, not only a passive epigenetic event. But the underling mechanism and cellular functions of HAND2 in cancer are unknown.

HAND2, a transcriptional factor, its reconstitution leads to perturbed the activation of MAPK/ERK signaling by reduction of ERK phosphorylation in this study. MAPK/ERK signaling pathway is crucial in growth factor stimulation and oncogenic potential in CRC [17]. ERKs require phosphorylation for activation in regulating cytoplasmic and nuclear targets [18]. In this study, we found HAND2 reconstitution could bind to ERK and reduce its phosphorylation for ERK inactivation to inhibit tumor growth. In previous study, loss of PTEN, that are frequently mutated and suppressed in tumors, can be induced in HAND2 knock-out mice [8]. However, loss of PTEN expression in HAND2 reconstitution cells was shown in mRNA level but not observed in protein level, which need further confirmation.

Our data have two potential major implications. Firstly, epigenetic alterations, such as HAND2 methylation, are important functional event during early tumorigenesis and not just cancer passive characteristics. Although HAND2 are found to act crucial roles in development, mainly focusing on regulating downstream targets as one transcription factor [12, 13, 19], only one recent study reveals epigenetic silencing roles of HAND2 in early carcinogenesis of endometrial cancer [8], it remains unclear whether HAND2 methylation has functional relevance in other types of cancers. In our study, we firstly identify HAND2 methylation is a frequent epigenetic event in CRC and is functionally relevant to gene silencing. The downstream classic MAPK/ERK signaling of HAND2 is clarified and HAND2 acts as a tumor suppressor in CRC.

Secondly, CRC is the third most common cancer, but over 50% of CRC tumors will develop metastasis and cause poor outcomes, while early stage CRC can obtain long survive time. Current screening tools for early detection of CRC, mainly including colonoscopy and stool-based fecal immunochemical tests, are limited in many aspects, such as the invasiveness and specificity. Epigenetic cancer specific alterations, especially in driver genes, are often early molecular events in many types of cancers [3, 8]. The potential clinical applications of HAND2 methylation are significant in early detection of CRC and in stratifying subtypes of CRC outcomes, and also are promising in developing more epigenetic targeted therapies.

Despite these notable findings, there are still some limitations in this study: (1) other potential targets of HAND2 such as PTEN signaling, and EGFR-RAS signaling need to be clarified; (2) the mechanism of HAND2 in binding to ERK and changes of downstream key molecules in ERK signaling need to be clarified in future study; (3) clinical application of HAND2 methylation and expression in early detection of CRC needed prospective trials. Thus, further studies addressing these limitations are needed to provide more evidences of the roles of epigenetic HAND2 alteration in CRC.

## Conclusions

HAND2 is one tumor suppressor by targeting ERK signaling and one potential epigenetic driver gene in CRC.

## Methods

### Tissue samples and patients

Fresh frozen tissues of colorectal cancers and paired normal control tissues were obtained from the Sixth Affiliated Hospital of Sun Yat-Sen University (SYSU) in Guangzhou, China. This study followed local ethical protocol. One pathologist reviewed all of these tumor tissues to confirm the diagnosis. All fresh tissues were stored under − 80 °C. The tumor samples and the clinical-pathological parameters were collected from a well-run electronic CRC database of this hospital.

Informed consent was obtained from all patients enrolled into this CRC database. TNM stage was defined according to the 6th version of American Joint Committee on Cancer (AJCC) staging Manual. Follow-up was conducted according to the guideline of National Comprehensive Cancer Network (NCCN). The overall survival (OS) was defined as the time interval from radical surgery to death. The study was approved by Human Medical Ethics Committee of SYSU.

### Cell culture

Human CRC cell lines (DLD1, RKO, SW480, HCT15 and HCT8) were all cultured in RPMI-1640 or DMEM medium (Gibco, USA), supplemented with 10% FBS and 1% penicillin and streptomycin (Invitrogen, USA) in the humidified incubator at 37 °C with 5% CO_2_. All cell lines were purchased from American Type Culture Collection (ATCC).

### Cell proliferation, invasion and migration

Cell proliferation was assessed by both real-time cellular analysis (RTCA) DP device (ACEA biosciences, USA) and cell counting kit-8 (CCK-8, Dojindo lab, Japan) as our previous studies [20, 21]. Cells are plated in 96-well plate at 6000–8000 cells per well and CCK-8 was added at appointed time and incubate for 2 h and the optical density was measured by multimode spectrum system (Thermo, USA) at 450 nm. In RTCA assays, optical density of cells was seeded and automatically monitored every 15 min.

Cell invasion and migration were assessed by transwell assays with 8 um pore size of cell culture insert (Corning, USA) with or without Matrigel according to the manufacturer’s protocols as our previous studies [20, 21]. Appropriate density of cells were plated onto membrane coated with (invasion) or without (migration) Matrigel and fibronectin in the upper chamber of 24-well insert that contains serum free medium. The bottom chamber contained growth medium with 20% FBS. Cells were incubated for 48 h and then cells on the bottom of upper chamber insert were fixed by paraformaldehyde and stained with crystal violet, and images were captured on inverted microscope. All of above assays were repeated at least three times.

### DNA, RNA extraction

Genomic DNA (gDNA) was extracted from fresh frozen tissues after thawing by DNeasy Blood and Tissue Kit (Qiagen) according to manufacturer’s instructions. After extraction, gDNA was diluted into a total volume of 100ul and was quantified with a ND-100 spectrophotometer (NanoDrop technologies). Diluted DNA was bisulfited and converted using EZ DNA methylation Kit (Zymo Research) following the manufacturer’s instructions.

Total RNA extraction and reverse transcription was described previously [20, 21]. Total RNAs were extracted using TRIzol reagent (Invitrogen) according to the manufacturer's instructions. Reverse transcription was conducted with Revertra Ace PCR RT master mix with gDNA remover (Toyobo, Japan).

### Real-time qPCR

The qPCR assays were conducted by using Taqman methods according to the instructions. The designed primers and probes of target genes and iTaq Universal Probes Supermix were purchased (Applied Biosystems, USA). The qPCR running conditions were as follows: 95 °C for 10 min; and 40 cycles (denaturation at 95 °C for 15 s, annealing/extension temperature at 60 °C for 1 min). All assays were performed in duplicates, including a negative control without template.

The sequences of primers and probes are reserved by the company, and can be ordered through Applied Biosystems website with the following order numbers: HAND2 (*Sc-25346, Santa Cruz; MAB8546, R&D*), EGFR (MA5-13070, Thermo Fisher), ERK 1/2 (Sc-514302, Santa Cruz), p-ERK (Sc-7383, Santa Cruz), PTEN (Sc-7974, Santa Cruz), Ras (3965, Cell Signaling). The housekeeping gene HPRT1(Santa Cruz, sc-376559) or Tubulin (Sc-73242, Santa Cruz) were applied as the endogenous reference gene. Relative gene expression was normalized to HPRT1 using the 2^−∆∆CT^ method. All Taqman qPCR assays were conducted by CFX96 Touch Real-Time PCR Detection System (Bio-Rad).

### Methylation PCR

Quantitative MethyLight PCR (qMSP) assays using iTaq Universal SYBR Green Supermix (Bio-Rad, USA) by CFX96 Touch Real-Time PCR Detection System (Bio-Rad, USA). The qMSP assays were conducted according to our previously studies [22, 23]. Briefly, the primers of HAND2 were firstly evaluated by end-point hemi-quantitative PCR. The PCR product was assayed by horizontal gel electrophoresis in 1.5% agarose gel and then the specificity of HAND2 methylated primers were identified. Bisulfite-converted DNA was loaded as templates for qMSP, including HAND2 primers, SYBR Green Supermix and water. 100% methylated and unmethylated EpiTect Methyl DNA standards (Qiagen) were used for positive and negative controls. The thermocycler conditions were as follows: 95˚C for 15 min followed by 45 cycles of 95˚C for 15 s and 60˚C for 1 min. Relative methylation percentage (RM%) was calculated as ratio of 100 X (methylation/control), in which methylation refers to the amount of methylated HAND2, while control refers to imput total bisulfite-converted DNA by ALUC4 MethyLight assay. All samples were run in duplicate assays. Data was analyzed by BioRad CFX manager software v3.1 and Cq was determined with Single Threshold method (Bio-Rad).

The primers sequences of HAND2 methylated site (cg02774429) were listed as follows. The probe of HAND2 is located in the + 127 position of promoter region, which was indicated in previous study [8].

Forward primer: CCTCTCCTTTCGAAACAAAAATCTAA;

Reverse primer: TTAGTTTAGGAGAATTATCGTCGTTATTTC.

The primer sequences of ALUC4 control, using to normalize input DNA amounts, were listed as follow:

Forward: GGTTAGGTATAGTTGTAATTTTAGTA;

Reverse: ATTAACTAAACTAACTCCTAACCTCA.

### SiRNA knockdown and overexpressed HAND2 plasmid

The siRNA of HAND2 and scrambled control (si-NC) were purchased from Ribo company (Guangzhou, China). Sequences of siRNAs were persevered by the company. The siRNAs were transfected into cells by Lipofectamine RNAiMax (Invitrogen) according to instructions as our previous study.

The overexpressed HAND2 plasmid was constructed by inserting HAND2 sequence from RACE assay into pcDNA3.1 + plasmid at multiple cloning site with KpnI and Xhol restriction enzymes (New England Biolabs) and was ligated using Ligation high kit (Toyobo, Japan). The constructed plasmids were transfected with cells by lipofectamine 3000 (Invitrogen) according to instructions as our previous study [21].

### Plasmid construction and lentiviral infection

Stable overexpression of HAND2 plasmid was constructed by ligating HAND2 to PCDH-GFP vector with in-fusion HD cloning kit (Clontech) according to manufacturer’s instructions as our previous study [21]. PCDH-HAND2 was co-transfected with pCMV-Δ8.91 and pCMV-VSVG into 293 T cells to generate viral supernatants by using lipofectamine 3000. The lentivirus was concentrated by cold high-speed centrifuge and targeted cells were infected with lentivirus by incubating together for 48 h. Overexpressed clones with gree lights of GFP were selected and confirmed by qRT-PCR.

### Western Blot

Cells were lysed by RIPA lysis buffer (Beyotime, China) and protein was extracted and measured by BCA protein assay kit (Beibo, China). Protein were denaturated by SDS-PAGE buffer, separated by 5% stacking gel and 10% running gel, and transferred to NC membranes (Millipore, USA). The blots were blocked with 5% non-fat milk with Tween-20 (TBST) and then incubated with primary antibody overnight and second antibody of HRP-labeled IgG for 1 h, and scanned by enhanced chemiluminescence system (Odyssey, USA). Image J software was used for quantitative analysis and proteins were normalized to internal control β-actin or Tubulin. The primary antibodies used were as follows: HAND2, GATA2, ERK, P-ERK, PTEN, RAS, Tubulin.

### Luciferase reporter assay

For luciferase reporter assay, pGL4-GATA2-promoter plasmid with promoter region of GATA2 was constructed. Then the plasmid was co-transfected in CRC cell lines with pRL-TK plasmid and pcDNA3.1-HAND2 overexpressed plasmid by Lipofectamine 3000. After incubating for 24-48 h, luciferase activity was then measured by Dual luciferase Reporter Assay System (Promega, E1910, USA) according to manufacturer’s instructions. The site mutation of pcDNA3.1-GATA2 in the promoter region site 1 (502–522 bp), site 3 (471–491 bp), and site 4 (1020–1040 bp) were performed with KOD-Plus-Mutagenesis Kit (TOYOBO, SMK-101) according to manufacturer’s instructions. Then co-transfection and Luciferase assays were conducted according to our previous study [21].

### Tumor xenograft and IHC analysis

For construction of CRC xenograft, 5 weeks old BALB/c nude mice were purchased from laboratory animal center of SYSU (Guangzhou) and were feed in SPF conditions. HAND2 overexpressed plasmid or control plasmid was stably transfected into DLD1 cells by lentivirus method. A volume of 4 × 10^6^ cells was subcutaneously injected into each BALB/c nude mice. After 24 days, all mice are euthanized by cervical dislocation and xenograft tumor were collected and weighted. Each group contained seven mice. Every 3–4 days, tumor size was recorded and calculated as volume = (length × width^2^)/2. Tumor tissue were fixed, wrapped and stained by anti-Ki67 according to routine IHC method. The details are described in our previous study [21].

### RNA-sequencing assays

Whole transcriptome deep sequencing (RNA-seq) was performed by Kangchen Biotech Company with Illumina Hiseq 4000 (Shanghai, China) as our previous study [21]. Four groups were designed, including paired DLD 1 siNC/siRNA and overexpressed paired DLD1 pcDNA3.1/HAND2OE. Heatmap analysis was performed with OmicShare tools (a free platform for data analysis, http://www.omicshare.com/tools). Gene Ontology (GO) analysis of RNA-seq data was performed by R software. Gene Set Enrichment Analysis (GSEA) were conducted with GSEA ranked tool analysis.

### Chromatin immunoprecipitation (CHIP)

For detection of HAND2 protein and GATA2 promoter DNA binding, CHIP was conducted by Chromatin IP kit (R & D systems). Crosslink the protein-DNA complexes by incubating CRC cells with 37% formaldehyde. Then sonicate the samples to shear chromatin, centrifuge the lysates and collect supernatant. HAND2 antibody, normal IgG, or RNA polymerase II were added to the samples and incubate in ultrasonic bath. Magnetic streptavidin beads were incubated with samples and washed. The samples were boiled with a temperature-controlled water bath, and centrifuge and supernatant were collected. DNA in the sample was extracted using DNA purification kit. Purified DNA was then prepared for PCR reactions.

### Statistical analysis

Student's t-tests or Chi-square test was performed appropriately for the comparisons. The correlation analyses between paired methylation and expression, correlation between expressions of different genes were performed by spearman rank correlation test. These tests were conducted using GraphPad Prism V5.01 (GraphPad Software Inc.). Potential risk factors of OS were evaluated by univariate analysis. These risk factors with *P* < 0.10 in univariate analysis were included in the subsequent multivariate analysis using Cox proportional hazards model. These above tests were performed by SPSS version 17.0 software (Chicago, IL, USA). All *P-*values reported were two-sided and the difference with a *P* < 0.05 was considered to be statistically significant.

## Supplementary Information

**Additional file 1. Supplement Figure S1**. Decreased HAND2 expression were observed in CRC tissues (0.958±1.474) of Chinese cohort 2, comparing to paired normal tissues (1.836±1.583) (n=86 pairs, *P*<0.001).

**Additional file 2**. **Supplement Figure S2** The methylation level of HAND2 were negatively correlated with mRNA expression in TCGA CRC cohort (n=247, R^2^=0.155, *P*<0.001). HAND2 methylation is calculated by beta value= Methylated probe intensity(M)/(Unmethylated probe intensity (U)+ Methylated probe intensity(M)+100) in the TCGA database.

**Additional file 3**. **Supplement Figure S3**. Cells with HAND2 reconstitution (pcHAND2) obtained no obvious changes of cell cycle compared to control vector (pcDNA3.1) in both DLD1 cells (A) and HCT8 cells (B) by FACS analysis.

**Additional file 4**. **Supplement Figure S4**. Increased PTEN expression were shown in cells with HAND2 reconstitution (HAND2OE), while no significant changes of KRAS, EGFR, TGFb2, SMAD4 or ERK were observed when comparing to control cells (pcDNA3.1) by qPCR assay to confirm RNA-seq profiles in both DLD1 and RKO cells.

**Additional file 5**. **Supplement Figure S5**. HAND protein expression was decreased in tumor tissue of CRC. We have added the microarray of IHC staining of HAND2 expression in the tumor tissue (lower expression) and normal colon tissue (higher expression) of CRC patients.

## Data Availability

All data generated or analysed during this study are included in this published article and its supplementary information files.

## Abbreviations

CRC: Colorectal cancer
HAND2: Heart and neural crest derivatives expressed 2
CHIP: Chromatin immunoprecipitation
GO: Gene Ontology
OS: Overall survival
qMSP: Quantitative MethyLight PCR
GSEA: Gene Set Enrichment Analysis
