# Correction: Epigenetic silencing and tumor suppressor gene of HAND2 by targeting ERK signaling in colorectal cancer

**DOI:** 10.1186/s12964-022-00944-x

**Published:** 2022-07-27

**Authors:** Zixu Yuan, Xihu Yu, Wenle Chen, Daici Chen, Jian Cai, Yingming Jiang, Xiaoxia Liu, Zhijie Wu, Lei Wang, William M. Grady, Hui Wang

**Affiliations:** 1 Department of Colorectal Surgery, Guangdong Provincial Key Laboratory of Colorectal and Pelvic Floor Diseases, Guangzhou, China; 2 Guangdong Institute of Gastroenterology, The Sixth Afliated Hospital of Sun Yat-Sen University, Guangzhou, China; 3 Clinical Research Division, Fred Hutchinson Cancer Research Center, 1100 Fairview Ave N, D4-100, Seattle, WA 98109 USA

## Correction to: Cell Communication and Signaling 20, 111 (2022) https://doi.org/10.1186/s12964-022-00878-4

Following publication of the original article [1], the authors identified a typesetting error in their article. The equal contributions statement was accidentally omitted from the published article.

† Zixu Yuan, Xihu Yu and Wenle Chen contributed equally to this study.

The original article [1] has been updated.
