# Supplementary figures and images for: Epigenetic silencing and tumor suppressor gene of HAND2 by targeting ERK signaling in colorectal cancer
Source: Cell Commun Signal. 2022 Jul 23;20:111. doi: 10.1186/s12964-022-00878-4 (PMC9308366; doi:10.1186/s12964-022-00878-4)

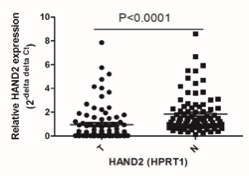

Supplement: Supplementary file 2 — Additional file 1. Supplement Figure S1. Decreased HAND2 expression were observed in CRC tissues (0.958±1.474) of Chinese cohort 2, comparing to paired normal tissues (1.836±1.583) (n=86 pairs, P<0.001). [file 12964_2022_878_MOESM2_ESM.jpg]

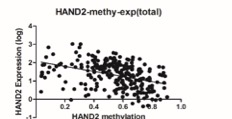

Supplement: Supplementary file 3 — Additional file 2. Supplement Figure S2 The methylation level of HAND2 were negatively correlated with mRNA expression in TCGA CRC cohort (n=247, R2=0.155, P<0.001). HAND2 methylation is calculated by beta value= Methylated probe intensity(M)/(Unmethylated probe intensity (U)+ Methylated probe intensity(M)+100) in the TCGA database. [file 12964_2022_878_MOESM3_ESM.jpg]

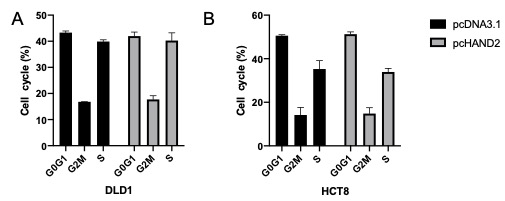

Supplement: Supplementary file 4 — Additional file 3. Supplement Figure S3. Cells with HAND2 reconstitution (pcHAND2) obtained no obvious changes of cell cycle compared to control vector (pcDNA3.1) in both DLD1 cells (A) and HCT8 cells (B) by FACS analysis. [file 12964_2022_878_MOESM4_ESM.jpg]

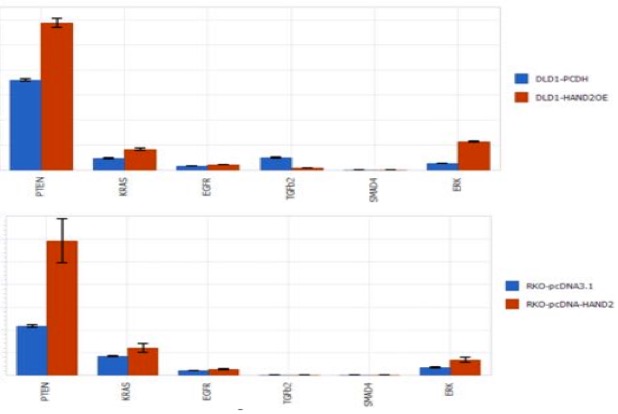

Supplement: Supplementary file 5 — Additional file 4. Supplement Figure S4. Increased PTEN expression were shown in cells with HAND2 reconstitution (HAND2OE), while no significant changes of KRAS, EGFR, TGFb2, SMAD4 or ERK were observed when comparing to control cells (pcDNA3.1) by qPCR assay to confirm RNA-seq profiles in both DLD1 and RKO cells. [file 12964_2022_878_MOESM5_ESM.jpg]

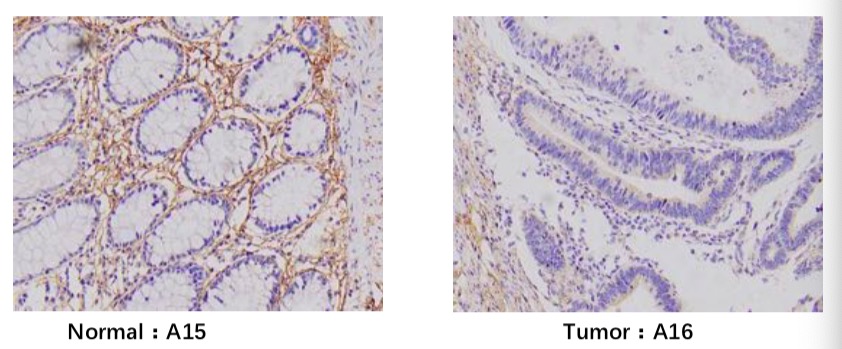

Supplement: Supplementary file 6 — Additional file 5. Supplement Figure S5. HAND protein expression was decreased in tumor tissue of CRC. We have added the microarray of IHC staining of HAND2 expression in the tumor tissue (lower expression) and normal colon tissue (higher expression) of CRC patients. [file 12964_2022_878_MOESM6_ESM.file]
